# The worldwide costs of dementia 2015 and comparisons with 2010

**DOI:** 10.1016/j.jalz.2016.07.150

**Published:** 2017-01

**Authors:** Anders Wimo, Maëlenn Guerchet, Gemma-Claire Ali, Yu-Tzu Wu, A. Matthew Prina, Bengt Winblad, Linus Jönsson, Zhaorui Liu, Martin Prince

**Affiliations:** a Aging Research Center, Department of Neurobiology, Care Sciences and Society (NVS), Karolinska Institutet and Stockholm University, Stockholm, Sweden; b Division of Neurogeriatrics, Department of Neurobiology, Care Sciences and Society (NVS), Karolinska Institutet, Stockholm, Sweden; c Centre for Research and Development, Uppsala University/County Council of Gävleborg, Gävle, Sweden; d King's College London, The Global Observatory for Ageing and Dementia Care, Institute of Psychiatry, Psychology and Neuroscience, Health Service and Population Research Department, London, UK; e REACH: The Centre for Research in Ageing and Cognitive Health, College of Life and Environment Sciences, Department of Psychology, University of Exeter, Exeter, UK; f Institute of Mental Health, Peking University, Beijing, China

**Keywords:** Dementia, Alzheimer's disease, Cost, Economics, Costs of illness

## Abstract

**Introduction:**

In 2010, Alzheimer's Disease International presented estimates of the global cost of illness (COI) of dementia. Since then, new studies have been conducted, and the number of people with dementia has increased. Here, we present an update of the global cost estimates.

**Methods:**

This is a societal, prevalence-based global COI study.

**Results:**

The worldwide costs of dementia were estimated at United States (US) $818 billion in 2015, an increase of 35% since 2010; 86% of the costs occur in high-income countries. Costs of informal care and the direct costs of social care still contribute similar proportions of total costs, whereas the costs in the medical sector are much lower. The threshold of US $1 trillion will be crossed by 2018.

**Discussion:**

Worldwide costs of dementia are enormous and still inequitably distributed. The increase in costs arises from increases in numbers of people with dementia and in increases in per person costs.

## Highlights

• The worldwide costs of dementia were estimated at United States (US) $818 billion in 2015.
• This estimated worldwide costs represent an increase of 35% since 2010.
• Eighty-six percent of the costs occur in high-income countries.
• Costs of informal care and the direct costs of social care still contribute similar proportions of total costs, whereas the costs in the medical sector are much lower.
• The threshold of US $1 trillion will be crossed by 2018.

## 1 Introduction

In 2010, Alzheimer's Disease International (ADI) presented estimates of the global societal economic impact of dementia [1], [2] also included in the World Health Organization/ADI 2012 joint report, “Dementia: a public health priority” [3]. The global cost in 2010 was estimated to be United States (US) $604 billion (bn). This figure equated to around 1% of the aggregated world gross domestic product (GDP), indicating a particularly significant global socioeconomic impact for this one disorder. Although most people with dementia live in lower middle-income countries (LMIC), almost 90% of the costs were incurred in high-income countries (HIC). The estimates of the likely prevalence of dementia have been updated for some regions since 2010, and the numbers affected have increased for all regions in line with the increase in the older population [4]. Cost of illness (COI) estimates have improved, with more recent and comprehensive studies carried out across the world. Thus, it is timely to update the global estimates of the economic impact of dementia. This article summarizes the major findings of the global COI estimates in the World Alzheimer Report of 2015 [4].

## 2 Methods

### 2.1 General approach

The current estimates of the global societal economic cost of dementia have been generated using the same general approach as for 2010 [2]. Costs are estimated at the country level and then aggregated in various combinations (worldwide cost, by World Bank [WB] country income level, by Global Burden of Disease world regions, and cost for G7 and G20 countries). For each country, there is a cost per person (per capita) estimate, which is then multiplied by the number of people estimated to be living with dementia in that country. The costs are divided into three cost subcategories: direct medical costs, direct social care costs, and costs of informal care.

The new estimates for 2015 should be considered to be a partial update of the 2010 estimates, rather than a full-scale revision. Regarding the numbers affected by dementia, this is based on a fully systematic updated review of prevalence studies [4]. We did not carry out a fully systematic review of COI studies. We identified several important COI studies published since 2010 (and used these to replace older COI data). We have included new cost estimates from the USA [5], UK [6], Germany [7], Norway [8], Sweden [9], and Ireland [10]. For low- and middle-income countries (LAMIC), there is more information available regarding costs of dementia care from seven countries surveyed by the 10/66 Dementia Research Group: China, India, Cuba, Peru, Venezuela, Dominican Republic, and Mexico (PhD thesis by Liu [11]).

As in 2010, for countries with no cost data, cost estimations are derived by imputation [1]. The assumption for the imputation is that there is a relationship between a country's per capita GDP and annual per capita direct costs of dementia. In the 2010 report, for LAMIC, the partitioning of the imputed total direct costs into direct medical and social care sector costs was derived from one Chinese study (Wang et al. [12]), where two-thirds of the direct costs were medical and one-third derived from the social care sector. These proportions were used as a basis for imputation in many Asian and African countries. Now, there is more information available from the 10/66 COI studies (China, India, Cuba, Peru, Venezuela, Dominican Republic, and Mexico) [11], where the proportions are similar to those from Wang et al. [12], but with a somewhat higher proportion of medical care costs in Latin America (74% of direct costs). Thus, the presumptions for imputations in LAMIC have improved considerably. Equivalent data from Africa are still lacking; therefore, we used the same principles for imputation as in the 2010 estimates.

For the 2010 cost estimates, there was only one published COI study from Latin America [13], which was used for imputation of estimates across the region. The thesis of Liu [11] has broadened the available information from Latin America considerably, making the imputations much more representative. The correlation between GDP per capita and annual direct costs of dementia per person in the updated set of COI studies used in the current report is 0.86 (*P* < .001).

### 2.2 Updating cost estimates from 2010 to 2015

For the current estimates, all costs are expressed as 2015 US dollars. The International Monetary Fund/World Economic Outlook (IMF/WEO) database of consumer price indices (CPIs) was used to generate cost adjustments, between 2010 and 2015, for each country [14]. For countries where no such figures were available, imputations based on trends from 2010 to the latest available CPIs were used. For a few countries with very small populations and not included in the WEO database, United Nation country profiles were used [15]. Such imputations were not required for any country with a major impact on the costs.

Two other issues are also important when interpreting comparisons between 2010 and 2015 costs. First, there have been shifts in the WB classification of country income level between 2010 and 2015 (several countries have been “upgraded”). To facilitate “like-for-like” comparisons between 2015 and 2010, the 2015 costs by country income level are presented according to a) the current 2015 WB classification and b) the 2010 WB classification. Second, the revised estimates of regional dementia prevalence arguably provide a better estimate of numbers of people with dementia in 2010 and 2015. For the World Alzheimer Report 2009 [16], we estimated 35.6 million people with dementia in 2010. However, if we apply the prevalence estimates from the current report, we would have estimated 40.1 million in 2010. The estimated numbers for China have increased considerably as have those for some countries in Northern Africa, whereas the estimates for some HIC (e.g., the USA and UK) are somewhat lower. The 2010 estimates based on the original prevalence estimates from the World Alzheimer Report 2009 are labelled in Table 1, Table 2, Table 3 as “WAR 2009,” whereas those based on the prevalence estimates from the current report are labelled as “WAR 2015.”

Using the trends (2010–2015) in per capita cost and numbers of people with dementia, each based on WAR 2015 prevalence, it is technically possible to make tentative forecasts of rates of future growth in costs. We present the estimated costs in 2030 and an estimate of the date when global cost will cross the threshold of US $1 trillion. To make a forecast of future trends in the global cost of dementia, we need to estimate trends in the numbers of people with dementia and trends in the per person costs. Trends per annum between 2010 and 2015 need to be estimated on a like-for-like basis. This means a) applying the WAR 2015 prevalence estimates to the 2010 and 2015 population structures to estimate numbers of people with dementia at both time points and b) using the same approach to weight the mean per capita costs.

### 2.3 Sensitivity analyses

Three sensitivity analyses have been included. In the 2010 report, the most significant effect in the sensitivity analysis was the method of quantifying informal care [1], [2]. In the main option, informal care is quantified in terms of time spent assisting with basic and instrumental activities of daily living (ADLs), whereas a lower cost (only basic ADLs) and a higher cost (basic and instrumental ADLs and time spent in supervision) are included.

The CPI is used for cost adjustments between 2010 and 2015 in the base option. In a second sensitivity analysis, the change in GDP in the different countries is used instead for the cost adjustments.

In a third “fixed-cost” sensitivity analysis, a crude prevalence-based alternative is presented, without any new COI data included and without cost adjustments. This sensitivity analysis focuses solely on the impact on costs of the changes in numbers of people affected.

## 3 Results

### 3.1 Aggregated costs

In the base option, the global costs of dementia have increased from US $604 bn in 2010 to US $818 bn in 2015 (Table 1), an increase of 35.4%. Our current estimate of US $818 bn represents 1.09% of aggregated global GDP. Excluding informal care costs, total direct costs account for 0.65% of global GDP; 86% of the costs occur in HIC.

The proportion of costs incurred in HIC is similar to that reported in the WAR 2010. Because many countries that were classified as low-income countries (LIC) or LMIC in 2010 have been “upgraded,” the proportion of worldwide costs incurred in upper middle-income countries (UMIC) has increased from 5.4% to 10.5%, and the proportion incurred in LIC and LMIC has decreased commensurately compared with 2010.

The effect of the WB reclassification of country income status is clearer if we compare 2010 and 2015 cost distributions, on a like-for-like basis, using the 2010 WB classification for both time points (Table 1). On this basis, the proportion of costs incurred in what were LIC and middle-income countries in 2010 has increased, and the proportion in what were HIC has decreased.

To complete the adjustments for a like-for-like comparison, we adjusted the 2010 COI estimates to take account of the revised prevalence estimates, which were used to estimate the 2015 costs (see Appendix 1, Supplementary Table 1). Despite the 4.9 million (14%) increase in the estimated numbers of people with dementia in 2010 when applying the WAR 2015 prevalence estimates, the total (worldwide) cost for 2010 increased marginally, from US $604.0 to 606.7 bn. The explanation for this is that most of the upward adjustments of numbers of people with dementia occurred in LIC and middle-income countries (where per capita costs are low), whereas there were some downward adjustments in the estimates of numbers of people affected in HIC where per capita costs are high. It is clear that there have been only modest changes in the distribution of costs by country income level.

The G7 countries have initiated and lead the “Global Action against Dementia.” In Supplementary Table 2, cost estimates for the G7 countries and the wider G20 group of nations are seen. This analysis reveals a striking concentration of global costs among the world's wealthiest nations. Although the G7 countries account for just over a quarter of global prevalence, over three-fifths of global costs are incurred in these seven countries. The G20 nations account for a remarkable 92% of global costs.

The pattern of distribution of costs between the three major subcategories (direct medical, social care, and informal care) has not changed substantially (Table 2). The proportional contribution of direct medical care costs is still modest, particularly in HIC. There is an increasing relative contribution of direct social care sector costs and a decreasing relative contribution of informal care costs with increasing country income level. Costs according to the Global Burden of Disease regional country classification are available as Supplementary Tables 3–5.

### 3.2 Annual costs per person with dementia

Direct comparison of costs per person by WB country income level is complicated both by the year of the WB classification (2010 vs. 2015) and the basis for prevalence estimates (WAR 2009 vs WAR 2015). The optimal like-for-like comparison uses the WB classification of 2010 and the WAR 2015 prevalence estimates for both the 2010 and 2015 (column 2 vs. column 4 in Table 3). According to each of the four approaches, per person costs increase steeply with country income status. Comparison of column 2 with column 3 illustrates that the reclassification “upward” of countries that are still poorer than most of those in the group that they join brings down the average per person cost for the high income–level group. Thus in 2015, according to the latest WB income level classification, there is now little difference in mean per capita cost between LIC and LMIC (column 3). According to the optimal like-for-like comparison (column 4 vs column 2), per person costs have increased at each of the 2010 country income levels between 2010 and 2015, but most markedly in what were, in 2010, UMIC.

In 2015, the mean cost per person with dementia was US $43,680 in G7 countries, US $20,187 in G20 countries, and US $6757 in countries that were members of neither G7 nor G20 (not shown in table).

### 3.3 Sensitivity analyses

Depending on how informal care is quantified, there is a great variability in worldwide costs, from US $651 bn (only basic ADLs) to US $1057 bn (all ADLs and supervision), but with little variation in distribution by country income level (Table 4).

If the change in per capita GDP is used to update costs from 2010 to 2015 (Supplementary Table 6), the total costs are somewhat higher than for the CPI base option (Table 1). The marked increase in estimated costs for UMIC had the most significant impact on worldwide costs.

If a prevalence-based option is used (holding the costs per person fixed and ignoring new COI data), worldwide costs increase by US $91.2 bn (15.1%) (Supplementary Table 7), suggesting that just under half of the US $214 bn increase in costs between the 2010 and 2015 WAR estimates are accounted for by increases in prevalence and numbers affected.

### 3.4 Forecasts beyond 2015

If we use the adjusted prevalence numbers for 2010, there would have been 40.1 million with dementia in 2010, and the numbers would have increased by 16.6% or by 3.3% per year. Between 2010 and 2015, the average worldwide cost per person (a weighted average across countries, calculated on a like-for-like basis) increased from US $15,122 to 17,483 per year (an increase of 15.6% or 3.1 % per year).

**Fig. 1:**
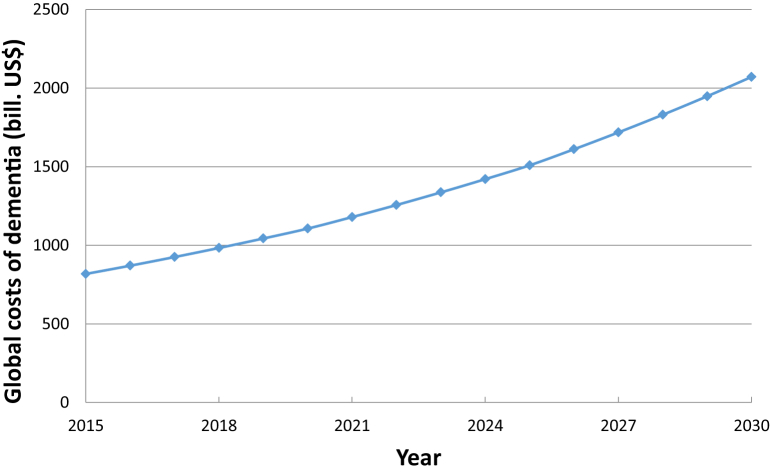
Forecasted global costs of dementia 2015–2030.

The overall annual trends can then be calculated as the product from increasing numbers (1.033) and increasing per capita costs (1.031) = 1.033 × 1.031 = 1.065 or around 6.5% per annum. By applying this figure, the costs in 2030 will be around US $2 trillion, and the threshold of US $1 trillion will be crossed in 2018 (Fig. 1).

## 4 Discussion

The global societal economic cost of dementia, US $818 bn, is an enormous sum, similar in magnitude to the GDP of countries like Indonesia, The Netherlands, and Turkey, the 16th to 18th largest economies in the world. The global costs are also larger than the market values of companies such as Apple (US $742 bn), Google (US $368 bn), and Exxon (US $357 bn) (source: *Forbes*, 2015 ranking).

As we reported in 2010, the costs remain concentrated in countries with higher income levels. There is an imbalance between the global distribution of prevalence, 58% of people with dementia living in LAMIC, and costs, 87% in HIC. This is accounted for by the lower per person costs in LAMIC, reflecting lower wage costs and a high proportion of care provided by informal unpaid carers. Costs when expressed as a proportion of GDP are certainly not negligible in LAMIC (ranging from 0.2 to 0.5%) but lower than those in HIC (1.4%). The uneven distribution of global costs is even more striking when stratified according to G7 (62 % of worldwide costs) or G20 membership (92% of global costs).

Our sensitivity analysis confirms that the assumptions regarding costing of informal care have a great impact on the total costs. Although difficult to quantify, supervision is an important and significant part of daily informal care with significant opportunity cost for carers. If that component is included, the costs increase considerably. Transparency regarding assumptions is crucial to make comparisons meaningful in any COI analysis.

Our current estimates of global societal costs of dementia have increased by around 35% compared with those published in the World Alzheimer Report 2010. Interpreting these increases is complex given the multiplicity of plausible underlying explanations whether it is because of increasing burden of dementia or because the methods for estimating the dementia burden has changed.

Increases in aggregated costs can arise from increases in numbers of people with dementia and/or increases in per person costs. The exploratory analyses that we have conducted suggest that these two elements each contribute around one half of the total increase. We have adjusted costs between 2010 and 2015 according to CPIs in each country. As developing economies grow, the cost of salaries and services tend to inflate more rapidly than prices (a “positive income elasticity effect”); so, this approach may have underestimated cost inflation in LAMIC relative to HIC, as indicated by our GDP-based inflation sensitivity analysis. In either scenario, cost inflation can have accounted for only part of the increase in per capita costs.

Per capita costs may also increase because of the following:

a) we have estimated them better, with more up-to-date studies,
b) some services have become more costly, and
c) new services have been established, the coverage of existing services has improved, or existing service users are using the same services more intensively.

We do not have adequate data to discern between these three sets of explanations. Some studies suggest that the proportion of people with dementia living in residential care has begun to decline in HIC, consistent with policy initiatives to provide care at home where possible [17]. However, such a strategy may not be associated with reduced costs, when all the costs of home care, including informal care, are properly accounted for [18]. It has also been suggested that cost-reduction initiatives may be reducing the intensity of home care (e.g., shorter and less frequent care worker visits) in the UK [6].

Economic development is proceeding apace in many LIC and, particularly, middle-income countries. This has posed a challenge for us in making meaningful comparisons between country income level groups for 2010 and 2015 because a significant number of countries, some of them very populous, have moved “upward” in the WB classification such as China, Bangladesh, Thailand, Iran, Russia, Poland, Kenya, and Argentina. The average cost per person with dementia in the higher WB groups is “diluted” by newly promoted countries with lower economic strength than the original countries in that particular WB group. At the same time, the remaining lower income countries are “drained” by the loss of more prosperous countries that have moved upward in the WB classification. We addressed this problem by stratifying the 2015 estimates according to the 2010 and the 2015 classification. The long-term care sector is very underdeveloped in most LAMIC, but economic growth, accompanied by social and demographic change, may increase demand resulting in the establishment and/or expansion of a formal long-term care sector as a complement to informal care.

Although the basics for the global cost estimates are available COI studies of dementia, the costing model imputes missing country data based on the assumed relationship between the economic strength of a country and resources for dementia care. COI studies from LAMIC are rare, with, therefore, a greater reliance on imputation for these countries. Nevertheless, the correlation between the GDP per capita and direct costs per person with dementia seems to be robust.

The current report is not a complete systematic update, although some important new COI studies are added and data on resource use and costs from the 10/66 Dementia Research Group. However, the number of cost components that are included in COI studies varies, which can make comparisons problematic. For example, in the new UK report, a cost estimate of people who had gone missing because of dementia was included, [6] and in the Swedish update, detailed costs of drug use were included [9]. The use of CPIs is not the optimal way of adjusting care costs. Price inflation indices specific to the health care and social care sector would be better, but such data are not yet available globally.

The cost forecasts should be treated with particular caution. Besides the generic heterogeneity of COI studies, we also had to make assumptions regarding the appropriate age-specific prevalence estimates to use at each time point. Furthermore, the dynamics of change in care patterns across regions and the potential for effective primary prevention programs for dementia are all hard to forecast.

It is our hope that more service utilization and COI studies will be carried out, improving the overall quality, coverage, and recency of the evidence base, which, coupled with an ongoing commitment to monitor trends in prevalence and numbers, will allow us to estimate global costs and trends with more accuracy. We are eager to integrate this work within plans for a Global Observatory to be coordinated by the World Health Organization.

**Research in context**

1. Systematic review: The estimated numbers affected by dementia are based on a fully systematic updated review of prevalence studies. We did not carry out a fully systematic review of cost of illness (COI) studies, but we identified several important new studies and used these to replace older data.
2. Interpretation: Worldwide costs of dementia are enormous and still inequitably distributed. The increase in costs arises from increases in numbers of people with dementia and in increases in per person costs.
3. Future directions: We hope that more service utilization and COI studies will be carried out, improving the overall quality, coverage, and recency of the evidence base, which, coupled with an ongoing commitment to monitor trends in prevalence and numbers, will allow us to estimate global costs and trends with more accuracy. We are eager to integrate this work within plans for a Global Observatory to be coordinated by the World Health Organization.

## Figures and Tables

**Table 1:** Worldwide costs of dementia in 2010 and 2015 (bn US dollars), based on WB country classifications 2010 and 2015

Basis for prevalence estimates	2010 (WAR 2009)			2015 (WAR 2015)			2015 (WAR 2015)		
WB country classification year	2010			2015			2010		
	Numbers of people with dementia (millions)	US dollars (bn)	Percent	Numbers of people with dementia (millions)	US dollars (bn)	Percent	Numbers of people with dementia (millions)	US dollars (bn)	Percent
LIC	5.0	4.4	0.7	1.2	1.2	0.1	7.0	6.6	0.8
LMIC	9.4	29.2	4.8	9.8	15.3	1.9	14.8	57.1	7.0
UMIC	4.8	32.5	5.4	16.3	86.3	10.5	8.1	84.5	10.3
HIC	16.4	537.9	89.1	19.5	715.1	87.4	16.9	669.6	81.9
Total	35.6	604.0	100.0	46.8	817.9	100.0	46.8	817.9	100.0

Abbreviations: bn, billion; HIC, high-income countries; LIC, low-income countries; LMIC, lower middle-income countries; UMIC, upper middle-income countries; WAR, World Alzheimer Report; US, United States; WB, World Bank.

**Table 2:** Subcategory costs of dementia in 2010 and 2015 (bn US dollars and percent of total costs), by country income level based on current WB country classification

	Direct medical costs		Direct social sector costs		Informal care costs	
	US dollars (bn)	Percent	US dollars (bn)	Percent	US dollars (bn)	Percent
2010 (WAR 2009)						
LIC	0.1	22.3	0.1	11.5	0.3	66.2
LMIC	2.9	29.4	1.6	16.4	5.3	54.2
UMIC	12.6	28.1	8.3	18.6	23.9	53.3
HIC	80.8	14.7	245.7	44.8	222.4	40.5
Total	96.4	16.0	255.7	42.3	251.9	41.7
2015 (WAR 2015)						
LIC	0.2	20.4	0.1	10.4	0.8	69.2
LMIC	3.7	23.9	2.0	13.2	9.6	62.9
UMIC	19.3	22.4	17.7	20.5	49.3	57.1
HIC	136.0	19.0	308.1	43.1	271.1	37.9
Total	159.2	19.5	327.9	40.1	330.8	40.4

Abbreviations: bn, billion; HIC, high-income countries; LIC, low-income countries; LMIC, lower middle-income countries; UMIC, upper middle-income countries; WAR, World Alzheimer Report; US, United States; WB, World Bank.

**Table 3:** Per person costs of dementia (US dollars) in 2010 and 2015, based on WB country classification (2010 or 2015) and prevalence estimates (WAR 2009 or 2015)

Column	2010		2015		Change (%) in costs per person (WAR 2015)
	1	2	3	4	
Year for cost estimates (basis for prevalence estimates)	2010 (WAR 2009)	2010 (WAR 2015)	2015 (WAR 2015)	2015 (WAR 2015)	
WB country classification year	2010	2010	2015	2010	2010
LIC	868	875	1019	939	7.3
LMIC	3109	3259	1560	3865	18.6
UMIC	6827	7224	5284	10,467	44.9
HIC	32,865	34,735	36,669	39,595	14.0

Abbreviations: HIC, high-income countries; LIC, low-income countries; LMIC, lower middle-income countries; UMIC, upper middle-income countries; WAR, World Alzheimer Report; US, United States; WB, World Bank.

**Table 4:** Sensitivity analysis

	Base option		More restrictive		More inclusive	
	All ADLs		Only basic ADLs		All ADLs and supervision	
	US dollars (bn)	Percent	US dollars (bn)	Percent	US dollars (bn)	Percent
LIC	1.2	0.1	0.9	0.1	1.6	0.2
LMIC	15.3	1.9	10.7	1.6	21.7	2.1
UMIC	86.3	10.5	75.0	11.5	121.2	11.5
HIC	715.1	87.4	564.9	86.7	912.2	86.3
Total	817.9	100.0	651.5	100.0	1056.8	100.0

Abbreviations: ADLs, activities of daily living; bn, billion; HIC, high-income countries; LIC, low-income countries; LMIC, lower middle-income countries; UMIC, upper middle-income countries; US, United States.

NOTE. Costs of dementia in 2015 (bn US dollars), by 2015 World Bank country income level, according to different approaches to costing informal care based on different caregiver inputs.
