# Supplementary material for: The worldwide costs of dementia 2015 and comparisons with 2010
Source: Alzheimers Dement. 2017 Jan;13(1):1–7. doi: 10.1016/j.jalz.2016.07.150 (PMC5232417; doi:10.1016/j.jalz.2016.07.150)
Supplement: Supplementary Tables 1–7 [file mmc1.docx]

Appendix 1

Supplementary tables.

*Supplementary table 1.* *Worldwide costs of dementia in 2010 and 2015 (billion US$), based on World Bank country classification 2010 and adjusted prevalence figures of 2010.*

| Year for cost estimates (basis for prevalence estimates) | 2010 (WAR 2015) | | 2015 (WAR 2015) | |
| --- | --- | --- | --- | --- |
| World Bank Country Classification Year | 2010 | | 2010 | |
|  | US$ (billions) | Per cent | US$ (billions) | Per cent |
| Low income | 5.2 | 0.9% | 6.6 | 0.8% |
| Lower middle income | 41.2 | 6.8% | 57.1 | 7.0% |
| Upper middle income | 49.4 | 8.1% | 84.5 | 10.3% |
| High income | 510.9 | 84.2% | 669.6 | 81.9% |
| Total | 606.7 | 100.0% | 817.9 | 100.0% |

*Supplementary table 2.*  *Costs of dementia in 2015 (billion US$), by G7 and G20 country classification.*

|  | 2015 (WAR 2015) | | | |
| --- | --- | --- | --- | --- |
|  | US$ (billions) | Per cent of costs | Numbers of people with dementia (millions) | Per cent of prevalence |
| G7* | 508.7 | 62.2% | 12.9 | 27.6% |
| G20^¥^ | 754.2 | 92.2% | 37.5 | 80.1% |
| G20 excluding G7 | 245.5 | 30.0% | 24.6 | 52.6% |
| Rest of the world (excluding G20) | 63.6 | 7.8% | 9.3 | 19.9% |
| World | 817.9 | 100% | 46.8 | 100% |

** G7 countries: Canada, France, Germany, Great Britain, Italy, Japan, and the United States*

*^¥^ G20 countries: Argentina, Australia, Brazil, Canada, China, France, Germany, India, Indonesia, Italy, Japan, Mexico, Russia, Saudi Arabia, South Africa, South Korea, Turkey, the United Kingdom and the United States. The EU is the 20th ’country’ in the G20, for the purposes of this analysis the remaining EU member countries (Cyprus, Austria, Belgium, Croatia, Czech Republic, Denmark, Finland, Greece, Ireland, Luxembourg, Malta, Netherlands, Portugal, Slovenia, Spain, Sweden, Poland, Romania, Slovak Republic, Bulgaria, Estonia, Hungary, Latvia, Lithuania) were allocated to the G20 group.*

*Supplementary table 3. Costs of dementia in 2010 and 2015 (billion US$, and percent of worldwide costs), by Global Burden of Disease world region classification.*

| Year for cost estimates (basis for prevalence estimates) | 2010 (WAR 2009) | | 2015 (WAR 2015) | | 2010-2015 |
| --- | --- | --- | --- | --- | --- |
|  | US$ (billions) | Per cent | US$ (billions) | Per cent | Per cent change |
| Australasia | 10.1 | 1.7% | 14.1 | 1.7% | 39.6% |
| Asia Pacific High Income | 82.1 | 13.6% | 109.9 | 13.4% | 33.9% |
| Oceania | 0.1 | 0.0% | 0.2 | 0.0% | 59.0% |
| Asia Central | 0.9 | 0.2% | 1.2 | 0.1% | 28.6% |
| Asia East | 22.4 | 3.7% | 42.9 | 5.2% | 91.7% |
| Asia South | 4.0 | 0.7% | 4.5 | 0.5% | 11.8% |
| Asia Southeast | 4.0 | 0.7% | 7.3 | 0.9% | 81.9% |
| Europe Central | 14.2 | 2.3% | 15.0 | 1.8% | 5.7% |
| Europe Eastern | 14.3 | 2.4% | 23.5 | 2.9% | 64.3% |
| Europe Western | 210.1 | 34.8% | 262.6 | 32.1% | 25.0% |
| North America High Income | 213.0 | 35.3% | 268.9 | 32.9% | 26.3% |
| Caribbean | 3.0 | 0.5% | 3.5 | 0.4% | 18.2% |
| Latin America Andean | 0.9 | 0.2% | 1.1 | 0.1% | 27.0% |
| Latin America Central | 6.6 | 1.1% | 15.9 | 1.9% | 140.8% |
| Latin America Southern | 5.1 | 0.8% | 10.1 | 1.2% | 98.7% |
| Latin America Tropical | 7.3 | 1.2% | 15.6 | 1.9% | 113.8% |
| North Africa / Middle East | 4.5 | 0.7% | 16.7 | 2.0% | 270.7% |
| Sub-Saharan Africa Central | 0.1 | 0.0% | 0.3 | 0.0% | 198.6% |
| Sub-Saharan Africa East | 0.4 | 0.1% | 1.5 | 0.2% | 267.4% |
| Sub-Saharan Africa Southern | 0.7 | 0.1% | 2.3 | 0.3% | 221.7% |
| Sub-Saharan Africa West | 0.2 | 0.0% | 0.8 | 0.1% | 298.6% |
| Total | 604.0 | 100.0% | 817.9 | 100.0% | 35.4% |

*Supplementary table 4. Costs of dementia in 2015 (US$ billions), by Global Burden of Disease region classification. Costs in cost categories. Percentages of each GBD region class costs.*

| Cost sub-category | Direct medical costs | | Direct social sector costs | | Informal care costs | |
| --- | --- | --- | --- | --- | --- | --- |
| GBD World region | US$ (billions) | Per cent | US$ (billions) | Per cent | US$ (billions) | Per cent |
| Australasia | 1.0 | 6.9% | 7.1 | 50.3% | 6.0 | 42.8% |
| Asia Pacific High Income | 7.0 | 6.3% | 56.4 | 51.3% | 46.5 | 42.4% |
| Oceania | 0.0 | 17.4% | 0.0 | 8.6% | 0.1 | 74.0% |
| Asia Central | 0.3 | 29.6% | 0.3 | 25.3% | 0.5 | 45.1% |
| Asia East | 2.2 | 5.2% | 10.2 | 23.8% | 30.5 | 71.0% |
| Asia South | 0.5 | 10.7% | 0.1 | 3.3% | 3.8 | 86.0% |
| Asia Southeast | 2.7 | 36.8% | 1.3 | 18.2% | 3.3 | 45.0% |
| Europe Central | 2.8 | 18.8% | 3.1 | 20.4% | 9.1 | 60.8% |
| Europe Eastern | 5.7 | 24.1% | 4.9 | 20.7% | 13.0 | 55.2% |
| Europe Western | 50.8 | 19.3% | 113.0 | 43.0% | 98.9 | 37.6% |
| North America High Income | 61.1 | 22.7% | 115.5 | 43.0% | 92.3 | 34.3% |
| Caribbean | 0.8 | 21.3% | 0.8 | 21.8% | 2.0 | 56.9% |
| Latin America Andean | 0.2 | 17.8% | 0.4 | 32.6% | 0.6 | 49.5% |
| Latin America Central | 6.2 | 39.2% | 5.5 | 34.3% | 4.2 | 26.5% |
| Latin America Southern | 2.8 | 27.8% | 2.6 | 25.2% | 4.8 | 47.0% |
| Latin America Tropical | 5.7 | 36.8% | 5.2 | 33.4% | 4.7 | 29.9% |
| North Africa / Middle East | 8.5 | 50.7% | 1.2 | 7.2% | 7.0 | 42.0% |
| Sub-Saharan Africa Central | 0.1 | 28.5% | 0.0 | 14.1% | 0.2 | 57.3% |
| Sub-Saharan Africa East | 0.3 | 20.8% | 0.2 | 10.3% | 1.0 | 68.9% |
| Sub-Saharan Africa Southern | 0.4 | 16.4% | 0.2 | 8.1% | 1.7 | 75.6% |
| Sub-Saharan Africa West | 0.2 | 22.8% | 0.1 | 11.3% | 0.5 | 66.0% |
| Total | 159.2 | 19.5% | 327.9 | 40.1% | 330.8 | 40.4% |

*Supplementary table 5. Costs of dementia in 2010 and 2015 (costs per person with dementia, US$, and percentage change from 2010 to 2015), by Global Burden of Disease regional classification.*

|  | 2010 (WAR 2009) | 2015  (WAR 2015) | Change (%) in per capita costs (2010-2015) |
| --- | --- | --- | --- |
| Australasia | 32,370 | 36,404 | 12.5% |
| Asia Pacific High Income | 29,057 | 30,206 | 4.0% |
| Oceania | 6,059 | 7,021 | 15.9% |
| Asia Central | 2,862 | 3,723 | 30.1% |
| Asia East | 4,078 | 4,397 | 7.8% |
| Asia South | 903 | 872 | -3.5% |
| Asia Southeast | 1,601 | 2,021 | 26.3% |
| Europe Central | 12,891 | 14,056 | 9.0% |
| Europe Eastern | 7,667 | 12,104 | 57.9% |
| Europe Western | 30,122 | 35,255 | 17.0% |
| North America High Income | 48,605 | 56,218 | 15.7% |
| Caribbean | 9,092 | 9,387 | 3.2% |
| Latin America Andean | 3,663 | 3,375 | -7.9% |
| Latin America Central | 5,536 | 10,349 | 86.9% |
| Latin America Southern | 8,243 | 13,448 | 63.2% |
| Latin America Tropical | 6,881 | 9,426 | 37.0% |
| North Africa / Middle East | 3,926 | 6,955 | 77.2% |
| Sub-Saharan Africa Central | 1,081 | 1,880 | 74.0% |
| Sub-Saharan Africa East | 1,122 | 2,120 | 89.0% |
| Sub-Saharan Africa Southern | 6,834 | 9,490 | 38.9% |
| Sub-Saharan Africa West | 969 | 1,482 | 53.0% |

*Supplementary table 6. Costs of dementia in 2010 and 2015 by World Bank country income level (billion US$ and percent of total costs), based on current World Bank country classification for each year and cost adjustments (2010-2015) based on change in per capita GDP in each country .*

|  | 2010 (WAR 2009) | | 2015 (WAR 2015, GDP based cost adjustments) | |
| --- | --- | --- | --- | --- |
|  | US$ (billions) | Per cent | US$ (billions) | Per cent |
| Low income | 4.4 | 0.7% | 1.1 | 0.1% |
| Lower middle income | 29.2 | 4.8% | 15.0 | 1.7% |
| Upper middle income | 32.5 | 5.4% | 182.4 | 21.0% |
| High income | 537.9 | 89.1% | 671.0 | 77.2% |
| World | 604.0 | 100.0% | 869.6 | 100.0% |

*Supplementary table 7. Costs of dementia in 2010 and 2015 (billion US$), based on World Bank country classification 2010.*

|  | 2010 (WAR 2009) | | 2015 (WAR 2015) | |
| --- | --- | --- | --- | --- |
|  | US$ (billions) | Per cent | US$ (billions) | Per cent |
| Low income | 4.4 | 0.7% | 6.1 | 0.9% |
| Lower middle income | 29.2 | 4.8% | 48.2 | 6.9% |
| Upper middle income | 32.5 | 5.4% | 57.8 | 8.3% |
| High income | 537.9 | 89.1% | 583.0 | 83.9% |
| World | 604.0 | 100.0% | 695.2 | 100.0% |
